# Development of an approach to forecast future takeaway outlet growth around schools and population exposure to takeaways in England

**DOI:** 10.1186/s12942-024-00383-6

**Published:** 2024-11-10

**Authors:** Bochu Liu, Oliver Mytton, John Rahilly, Ben Amies-Cull, Nina Rogers, Tom Bishop, Michael Chang, Steven Cummins, Daniel Derbyshire, Suzan Hassan, Yuru Huang, Antonieta Medina-Lara, Bea Savory, Richard Smith, Claire Thompson, Martin White, Jean Adams, Thomas Burgoine

**Affiliations:** 1 Department of Urban Planning, College of Architecture and Urban Planning, Tongji University, Shanghai, China; 2 Key Laboratory of Ecology and Energy-Saving Study of Dense Habitat (Ministry of Education of China), Tongji University, Shanghai, China; 3 MRC Epidemiology Unit, University of Cambridge School of Clinical Medicine, Cambridge, UK; 4 Great Ormond Street Institute of Child Health, University College London, London, UK; 5 Nuffield Department of Primary Care Health Sciences, University of Oxford, Oxford, UK; 6 Office for Health Improvement and Disparities, Department of Health and Social Care, London, UK; 7 Department of Public Health, Environments & Society, Faculty of Public Health & Policy, London School of Hygiene & Tropical Medicine, London, UK; 8 Department of Public Health and Sport Sciences, Faculty of Health and Life Sciences, University of Exeter, Exeter, UK; 9 School of Health and Social Work, University of Hertfordshire, Hatfield, UK

**Keywords:** Takeaway food outlets (“takeaways”), Fast-food outlets, Takeaway management zones around schools, Exclusion zones, Time-series forecast, Population-level exposure, Public Health.

## Abstract

**Background:**

Neighbourhood exposure to takeaways can contribute negatively to diet and diet-related health outcomes. Urban planners within local authorities (LAs) in England can modify takeaway exposure through denying planning permission to new outlets in management zones around schools. LAs sometimes refer to these as takeaway “exclusion zones”. Understanding the long-term impacts of this intervention on the takeaway retail environment and health, an important policy question, requires methods to forecast future takeaway growth and subsequent population-level exposure to takeaways. In this paper we describe a novel two-stage method to achieve this.

**Methods:**

We used historic data on locations of takeaways and a time-series auto-regressive integrated moving average (ARIMA) model, to forecast numbers of outlets within management zones to 2031, based on historical trends, in six LAs with different urban/rural characteristics across England. Forecast performance was evaluated based on root mean squared error (RMSE) and mean absolute scaled error (MASE) scores in time-series cross-validation. Using travel-to-work data from the 2011 UK census, we then translated these forecasts of the number of takeaways within management zones into population-level exposures across home, work and commuting domains.

**Results:**

Our ARIMA models outperformed exponential smoothing equivalents according to RMSE and MASE. The model was able to forecast growth in the count of takeaways up to 2031 across all six LAs, with variable growth rates by RUC (min–max: 39.4-79.3%). Manchester (classified as a non-London urban with major conurbation LA) exhibited the highest forecast growth rate (79.3%, 95% CI 61.6, 96.9) and estimated population-level takeaway exposure within management zones, increasing by 65.5 outlets per capita to 148.2 (95% CI 133.6, 162.7) outlets. Overall, urban (vs. rural) LAs were forecast stronger growth and higher population exposures.

**Conclusions:**

Our two-stage forecasting approach provides a novel way to estimate long-term future takeaway growth and population-level takeaway exposure. While Manchester exhibited the strongest growth, all six LAs were forecast marked growth that might be considered a risk to public health. Our methods can be used to model future growth in other types of retail outlets and in other areas.

**Supplementary Information:**

The online version contains supplementary material available at 10.1186/s12942-024-00383-6.

## Background

Neighbourhood food environments have the potential to shape dietary behaviours, body weight and health [1]. Neighbourhoods with an abundance of takeaway food outlets (“takeaways”), which sell hot food prepared away from the home and designed for consumption off premises, have been identified as contributing negatively to health outcomes [2–5]. As a result, takeaways have become the focus of public health intervention. For example, the urban planning system can be used to shape the spatial distribution of takeaways, which is a retail sector that has experienced consistently strong growth over recent decades [6]. Specifically, urban planners within local authorities (LAs) in England can modify future takeaway exposure through denying planning permission for new takeaways. In England, the most common form of takeaway planning intervention is takeaway management zones around schools. LAs sometimes refer to these as takeaway “exclusion zones”. Within these zones (e.g. within 400 m of a school boundary) new takeaways can be prevented from opening. In addition to limiting future exposure to takeaways around schools for children and young people, these management zones also have significant potential to shape adult population exposure to takeaways because of their broad geographic coverage. Where adopted, takeaway management zones around schools cover an average of around 17% of any given LA [7].

The impacts of these takeaway management zones on retail process outcomes have recently been evaluated. Their adoption in 35 LAs in England was associated with an overall reduction in the number of planning applications received for new takeaways, and an increase in the proportion of those applications being rejected [7]. These impacts were observed at two years post-implementation. Early evidence of medium-term impact is also emerging at up to six years post-implementation [8]. However, for a policy like this, which does not and cannot change the current food environment but can only change how the environment will evolve in future, it is necessary to look over a much longer period to understand the extent of its potential impact.

As policies have not been in place for long, it is not feasible to directly observe the long-term retail impacts of management zones. Instead, times-series modelling can be used to estimate potential longer-term effects. For example, we can use current growth trends in takeaways in absence of the intervention to forecast future growth. Such estimates could then be used to understand the long-term impact of the actual intervention, informed by empirical data. Elsewhere, for example in public health research, time-series methods have been extensively used to forecast disease incidence [9, 10] and demand for hospital services [11, 12], to aid effective planning and management. However, studies specifically projecting future growth in the number of takeaways do not currently exist. One study attempted to forecast the number of restaurants in US zip code areas based on sociodemographic projections [13]. However, the applicability of this approach to forecasting takeaway growth is constrained by geographically limited data availability and the forecast range of future demographic trends. A more effective method would involve forecasting based on historic trends and other local factors, such as urban/rural status, which influence retail trajectories.

Long-term estimates of intervention impact on takeaway retail numbers are also necessary in the estimation of associated health impacts. An understanding of these health impacts could play a critical role in supporting LA decision-making and the effective implementation of planning policies to address takeaways. However, to facilitate this, any forecast of future takeaway retail would need to be transformed into a measure of population exposure to takeaways. Research suggests that comprehensive exposures considering where people live, work and travel on a day-to-day basis, determines health outcomes [3]. Therefore, a robust measure of population exposure needs to consider these different domains. There is currently no such population model available in the UK to facilitate this.

In this study, we describe the data sources and methods used to forecast future growth in the number of takeaways around schools from baseline (approximately 2015) to 2031. We present these forecasts for future takeaway growth in six LAs across England with different urban/rural characteristics. We also describe the approach for assessing population-level takeaway exposure by LA. This study is part of a larger project aimed at evaluating the long-term health and societal impacts of takeaway management zones around schools in England. The forecasting model and exposure computation approach developed here will be used to estimate “business-as-usual” scenarios in LAs with management zones and offer a framework that could extend to forecast growth in other retail sectors and areas, supporting research and policy-making. However, these broader applications are beyond the scope of the current paper.

## Methods

**Fig. 1:**
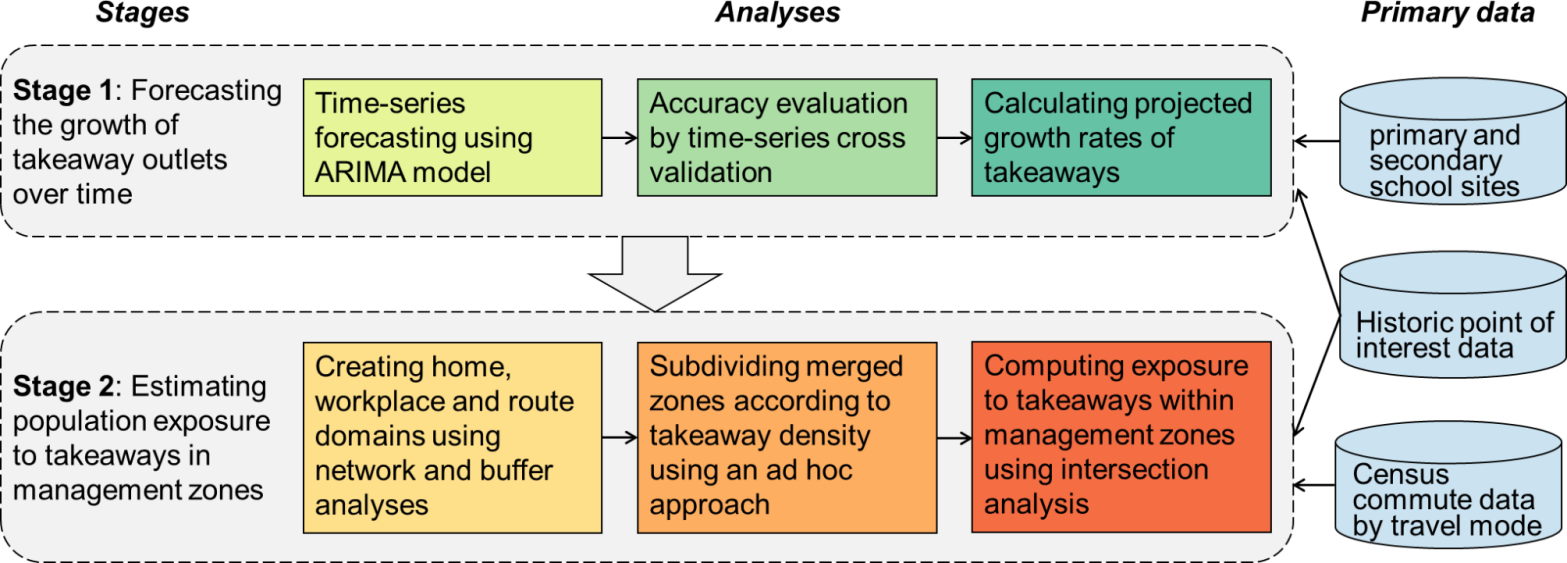
Key steps in the two-stage method to forecasting future takeaway growth and population exposure

We developed and validated a two-stage method to forecasting future takeaway growth and population exposure. First, we used a time-series model to forecast the future number of takeaways within management zones around schools within a small number of LAs. To maximise the accuracy of our forecasts, we learned from historic takeaway trend data in a national dataset of LAs that had not adopted takeaway management zones (hereafter referred to as non-adopter LAs). Second, we used these forecasts to estimate population exposure to takeaways within management zones in our small number of LAs by using census travel-to-work data (Fig. 1).

### Study area

#### Study region selection

Recognising that variations exist in baseline counts and historic growth rates of takeaways, as well as the possibility of contextually-specific influences on the impacts of intervention [7], we forecast future takeaway retail across a diverse range of LAs in England. We used the rural-urban classification (RUC) for LAs, which is based on the proportion of the total population living in urban settlements and conurbations [14]. The RUC describes LAs in England as belonging to one of five categories: “urban with major conurbation”, “urban with minor conurbation”, “urban with city and town”, “urban with significant rural” and “largely or mainly rural”. Additionally, we differentiated between LAs described as “urban with major conurbation” according to their location inside or outside of Greater London. We did this because population and travel behaviour characteristics, and the built environment including travel options, are likely to be different in Greater London compared to other major urban areas in England. Moreover, RUC with differentiation between London and non-London is often used as a comparative benchmark by policymakers at local and national levels in England [15], thereby enhancing generalisability of our findings.

**Fig. 2:**
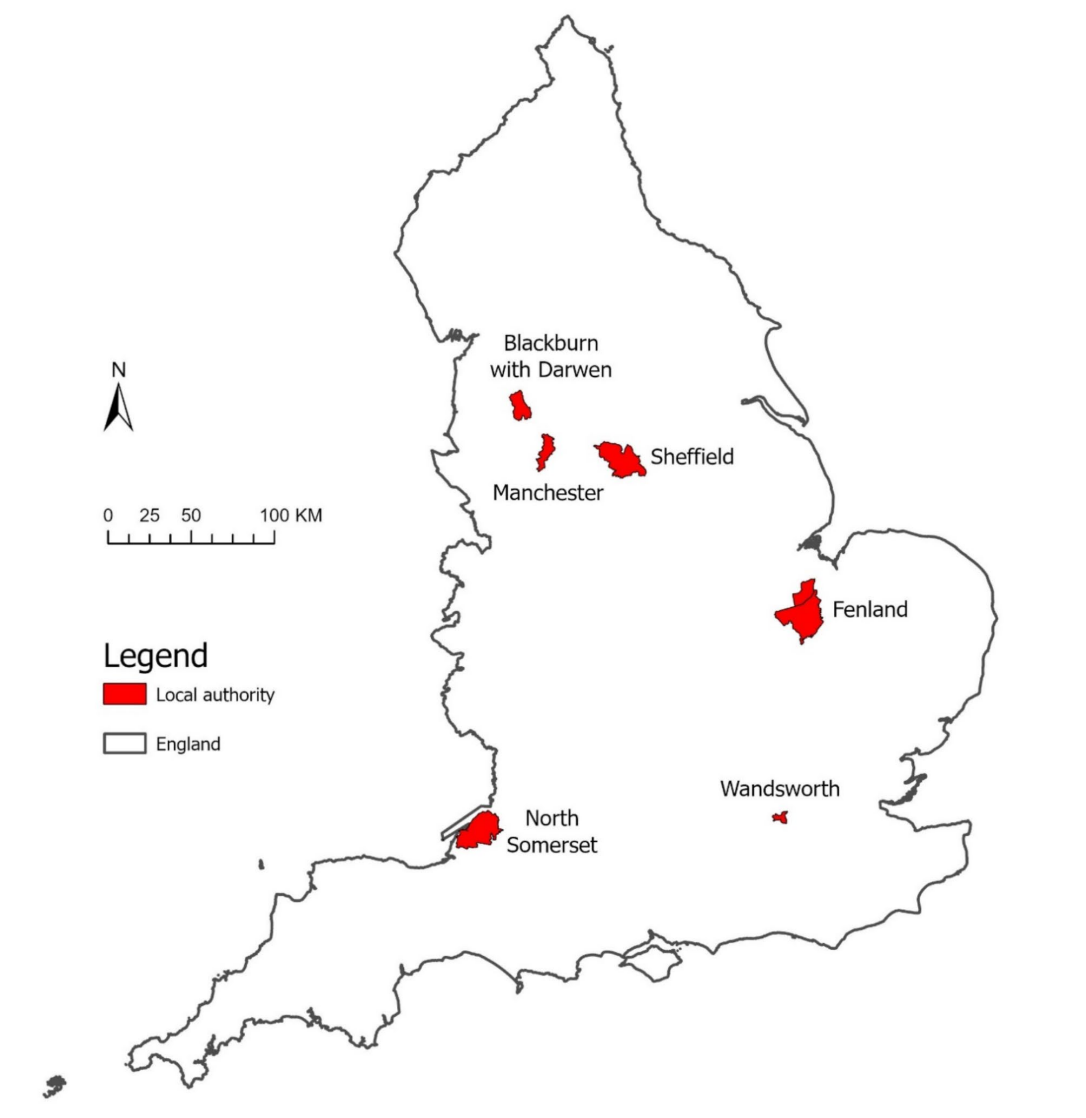
Map of the six selected adopter local authorities in England. Note: The base map of the boundaries of England was sourced from EDINA at the University of Edinburgh. © Copyright UK Data Service

One LA was purposively selected to represent each of the six RUC (Table 1), with consideration for maximizing geographical breadth across England (Fig. 2) and having similar years of policy adoption. Wandsworth, Manchester, and Blackburn with Darwen LAs (hereafter referred to as adopter LAs) were selected as they adopted the intervention around 2016. They had also applied management zones consistently around both primary and secondary schools. Sheffield, North Somerset, and Fenland LAs were further selected as *hypothetical* adopter LAs. They were selected to maximise geographic breadth and because there were no *adopter* LAs within their respective RUC classes. It was important to forecast growth in these types of LAs to allow for health impact modelling in rural areas, which could benefit from, but are not currently the focus of takeaway management zone interventions. In line with adopter LAs, these hypothetical adopters (hereafter also referred to simply as “adopter LAs”) were treated as if having adopted takeaway management zones around both primary and secondary schools in 2016.

**Table 1:** Classification of the six selected adopter local authorities across the rural/urban spectrum in England

Rural urban classification	Local authority	Adoption year	Baseline year^3^
London urban with major conurbation	Wandsworth	2015	2013
Non-London urban with major conurbation	Manchester	2017	2015
Urban with minor conurbation	Sheffield	“as if” 2016^1,^^2^	2014
Urban with city and town	Blackburn with Darwen	2016	2014
Urban with significant rural	North Somerset	“as if” 2016^1,^^2^	2014
Largely or mainly rural	Fenland	“as if” 2016^1,^^2^	2014

Notes:

1. Hypothetical adopter local authorities were chosen because no actual adopter local authorities existed before 2019 in their respective rural urban classification categories

2. In line with actual adopters, hypothetical adopter local authorities were treated as if having adopted takeaway management zones in 2016

3. The baseline year is set as two years prior to adoption to mitigate potential effects from the intervention’s announcement before its formal implementation

#### Management zones around schools

The most common form of takeaway management zones around schools adopted across LAs in England was a 400 m buffer extending from the boundary of school sites [7]. We defined management zones consistently in this way across all six LAs to permit a comparison of future forecast takeaway growth between LAs of different RUC. To recreate the boundaries of management zones in our six adopter LAs, and all other non-adopter LAs, polygons of school sites were obtained by querying primary and secondary schools from the OS MasterMap Sites Layer [16]. We eliminated double counting in overlapping areas among individual management zones and excluded takeaways outside the LA administrative boundaries, applying this approach consistently across both adopter and non-adopter LAs.

### Stage 1: Forecasting the growth of takeaway outlets over time

In the first stage, we developed a forecasting model to project the growth of takeaways within management zones. Our approach is based on the assumption that the business-as-usual growth trend in the six selected adopter LAs would mirror the projected trends of similar non-adopter LAs. To implement this, we used historic data from non-adopter LAs to build the forecasting model, which then generates growth rates that can be applied to project the business-as-usual trends in the adopter LAs.

#### Takeaways data

In this study, takeaways are defined as food outlets selling hot food intended for consumption off the premises. This definition aligns with use class A5 within the urban planning system in England. Takeaway management zones are designed to target A5 hot food takeaway outlets [17]. Information on the geographic locations of takeaways were sourced from Ordnance Survey (OS) Point of Interest (POI) data, which is an accurate, historic, and nationwide source of secondary information on the locations and types of food outlets in England [18]. Identification of takeaways was performed as described previously [8]. Briefly, takeaways were outlets within “fast food and takeaway outlets” (01020018), “fish and chip shops” (01020020) and “fast food delivery services” (01020019) OS POI categories [19]. Chain fast-food outlets, which commonly offer seating facilities, and those that primarily sell cold food items (e.g., sandwich shops) are exempt from takeaway management zones as they are not use class A5 within the urban planning system in England. To ensure consistency with the intervention of interest, these outlets were removed from the analytical POI dataset using string matching techniques [8].

Using OS POI data, we calculated the number of takeaways within these management zones, per quarter of calendar year, by intersecting zone geometries with takeaway locations in PostGIS [20]. The OS POI data were available quarterly from June 2011 to June 2021, but was missing in September 2011, December 2013, and March 2014. For these three quarters, the number of takeaways was imputed using linear interpolation.

#### Forecasting assumptions and approach

**Fig. 3:**
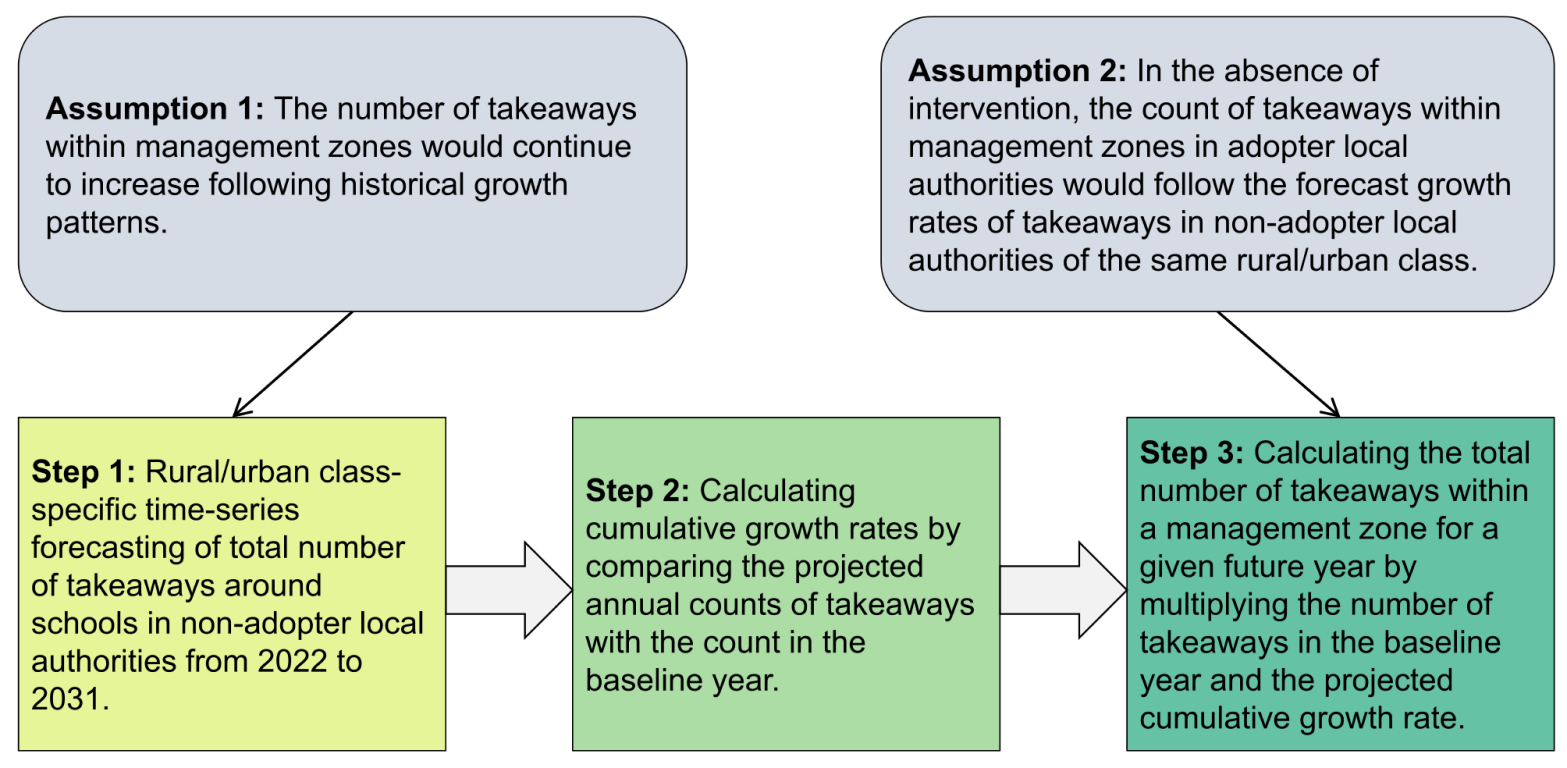
Illustration of our approach to forecasting counts of takeaways within management zones in adopter local authorities, including key assumptions made

Our forecast of future takeaway counts within management zones is based on two key assumptions (Fig. 3). First, the number of takeaways within these zones would continue to increase following historic pre-intervention growth patterns. However, the limited availability of past observations from within study LAs specifically, posed a challenge in developing a robust forecast model. Second, to overcome this challenge, we further assumed that in the absence of the intervention, counts of takeaways within management zones in our six LAs would follow the forecast growth rates observed for the same areas around schools in all other LAs of the same RUC in England (2011–2021), who had not adopted the intervention.

Based on these two key assumptions, we devised an approach to forecast counts of takeaways within management zones. The first step involved forecasting the total annual number of takeaways within management zones to 2031 in all non-adopter LAs using separate models for the six RUC classes. The forecast total counts of takeaways, along with their associated forecast intervals, are derived from a time-series model described in the next section.

The second step involved the conversion of forecast counts into cumulative growth rates. This conversion involves comparing the count of takeaways for any given future year to the count in the baseline year. We defined the baseline year as two years before the adoption of the intervention (i.e., 2013 for Wandsworth, 2015 for Manchester, and 2014 for other LAs) to exclude potential influences resulting from the announcement of the intervention before formal adoption.

Finally, the total number of takeaways within a management zone for a given future year is calculated by multiplying these two variables: the number of takeaways present within the management zone in the baseline year and the forecast RUC-specific cumulative growth rate corresponding to that future year.

#### Time-series forecasting using ARIMA model

Quarterly counts of takeaways within management zones were created to form a time series in which a sequence of observations are equally distributed in chronological order. The patterns within this series can be modelled using Auto-Regressive Integrated Moving Average (ARIMA) models, which are widely used in epidemiological studies [10]. ARIMA models allow estimation of future values by considering a linear combination of past values (i.e., the autoregressive (AR) process) and previous forecast errors (i.e., the moving average (MA) process). To apply ARIMA modelling may require differencing (i.e., the reverse of integration (I)) to eliminate any trend in the original time series [21]. For example, first-order differencing involves subtracting the previous observation (t-1) from the current observation (t) to create a new series. The full ARIMA model can be expressed as:

$$\begin{gathered}\:y_t^{\prime \:} = c + \:{\Phi _1}y_{t - 1}^{\prime \:} + \ldots \: \hfill \\\,\,\,\,\,\,\,\,\, + {\Phi _p}y_{t - p}^{\prime \:} + {\theta _1}{\varepsilon _{t - 1}} \ldots \: + {\theta _q}{\varepsilon _{t - q}} + {\varepsilon _t} \hfill \\ \end{gathered}$$

where $\:{y}_{t}^{{\prime\:}}$ represents the differenced series, and the terms on the right-hand side include a constant, lagged values of $\:{y}_{t}^{{\prime\:}}$, lagged errors, and the error term at time point $\:t$. This is referred to as an ARIMA(p,d,q) model, where d is the degree of differencing involved, and p and q are the orders of the autoregressive and moving average parts, respectively.

We fitted ARIMA models following established modelling procedures [21, 22], to takeaway count data from June 2011 to June 2021 in non-adopter LAs by RUC. The first step involved plotting the data to identify any unusual observations or signals of changing variance over time. In cases where variance exhibited temporal variation, we stabilized this using a Box-Cox transformation. In the second step, we administered the Kwiatkowski-Phillips-Schmidt-Shin (KPSS) test [23], which is a type of unit-root test used to identify whether a time series is stationary (i.e. where statistical properties do not depend on the time at which the series is observed). Parameter *d* was determined based on the degree of first-order differencing required to achieve stationarity. The third step involved exploring combinations of *p* and *q* values (p, q = 0, 1, 2, 3, 4, 5) on the stationary time series, in order to find the best model based according to bias-corrected Akaike information criterion (AICc). This is a modified version of AIC suitable for small sample sizes [24]. In the fourth step, we checked for autocorrelation in the residuals of the chosen model using a Ljung-Box test and the ACF plot of residuals. Finally, we calculated forecasts using the selected ARIMA model. The ARIMA modelling was conducted using R packages forecast [25, 26] and urca [27].

#### Time-series cross validation and accuracy metrics

In assessing the performance of these models, we implemented a time-series cross-validation procedure [22]. For this process, the time series was bifurcated into a sequenced collection of training and testing sets. Every individual observation was treated as a distinct test set, with the corresponding training set consisting of antecedent observations. The observations comprising the test sets are regarded as “forecasts”, and hence, the cross-validation does not involve any projected values. The initial two years of observations (*n* = 8) were excluded from test sets due to the limitations of acquiring a reliable forecast from a small amount of training data. Cross-validation was executed for horizons reaching up to 8 time points, which was equivalent to two years.

Forecast accuracy was captured as an average over all test sets. The metrics used for comparing accuracy were Root Mean Squared Error (RMSE) and Mean Absolute Scaled Error (MASE). MASE compares the mean absolute error of the forecast values using the current model to that of a one-step naïve forecast, in which forecasts are simply equal to the last observed value [28]. It avoids problems seen in other scale-free error metrics (e.g., undefined results of Mean Absolute Percentage Error (MAPE)) when one or more data point equals zero [10]. Smaller RMSE and MASE values indicate better model forecasting performance. The formulae are as follows:

$$\:RMSE = \sqrt {\frac{1}{n}\sum\limits_{i = 1}^n {{{({y_i} - {{\hat y}_i})}^2}} }$$

$$\:MASE = \frac{1}{n}\sum\limits_{i = 1}^n {\frac{{\left| {{y_i} - {{\hat y}_i}} \right|}}{{\frac{1}{{T - 1}}\sum\limits_{t = 2}^T {\left| {{y_t} - {y_{t - 1}}} \right|} }}}$$

where in both, *n* represents the number of forecasts, *T* represents the total number of observations in the time series, $\:{y}_{i}$ and $\:{\widehat{y}}_{i}$ denote the observed and predicted value of the *i*th forecast, and $\:{y}_{t}$ denotes the observed value at time point t.

Exponential smoothing (ETS), a well-established and widely used time-series forecasting method, was chosen for a sensitivity analysis (see Additional file 1). ETS is known for its simplicity and robustness in handling various types of time-series data, especially when dealing with trends and noise, as seen in the observed growth of takeaways over time in our data. As a widely recognized and accessible method, ETS provides a reliable benchmark for comparison against more complex ARIMA models [22]. However, ETS did not perform as well as ARIMA according to RMSE and MASE accuracy metrics in this study (see Additional files 1 and 2).

### Stage 2: Estimating population exposure to takeaways in management zones

We used forecast counts for six LAs to characterise population-level takeaway exposure by LA. We defined population exposure to takeaways as the total number of takeaways within management zones (i.e. the areas where takeaway exposure was liable to change in response to the policy) and in close proximity to the home, work, and commuting routes of the LA population. This measure of exposure across these three domains is consistent with previous UK work linking takeaway access to takeaway-type food consumption and body weight [3, 29].

#### Travel to work data

To model population exposure to takeaways, we acquired aggregated data on the adult population in employment aged 16 years and over, including commuting flows and modes from the 2011 UK Census. Specifically, we used population-level data on the number of commuters between residential and workplace output area (OA) pairs [30]. OAs are the lowest level of census geography, typically containing between 100 and 625 people [31]. In this context, a workplace is defined as the location where respondents reported typically working the most hours. We also obtained information on usual commuting mode (i.e., travel mode used for the longest part, by distance, of usual journey to work), which was reported as: car or van, motorcycle, tram/underground, bus, bicycle, or on foot [32]. Commute mode data was available at the lower super output area (LSOA) level, which is a spatial unit created by aggregating smaller OAs. Consequently, we assumed that the distribution of commuting modes in LSOAs is shared by OAs nested within, when estimating the number of commuters using a specific travel mode.

#### Creating home, workplace and commuting route exposure domains

Following previous research [3, 29], we used a 1-mile radius buffer to define home and work neighbourhoods. These buffers were centred on the population-weighted centroids of residential and workplace OAs [33]. Travel routes between home and work for those who reported using automobiles (i.e. car, motorcycle) and active transportation modes (i.e. cycling, walking) were modelled as the shortest connecting street network routes, using the Integrated Transport Network (ITN) provided by Ordnance Survey. For those who reported using public transport (i.e. bus, train), we assumed that no exposure occurred while on board [34]. However, shortest street network routes connecting homes and workplaces to their nearest bus stop or tram/underground entrance (i.e. ingress and egress trips) were modelled using transport network access point data from the National Public Transport Data Repository (NPTDR) [35].

Buffers of 500 m were applied along commuting routes for automobile travel, and 100 m for active transportation modes, in order to account for differing levels of accessibility to takeaways by travel mode. Ingress and egress trips were buffered by 100 m, as walking (i.e. active transport) to transit stops is common [36]. Home, work and commute route exposures were summed as overall exposure to takeaways across these three domains. Where individuals reported living and working within the same OA, or reported working from home, exposure within the home buffer was double-counted.

#### Deriving non-overlapping zones with distinct densities of takeaways

In densely populated areas, primary and secondary schools are often in close proximity, resulting in overlapping management zones. This overlap could lead to an overestimation in exposure since unique takeaways might be counted multiple times. Merging overlapping zones into a singular, unified feature could result in an excessively large polygon, which might mask the heterogeneous distribution of takeaways. Consequently, this approach might result in misclassification of takeaway exposure with management zones.

**Fig. 4:**
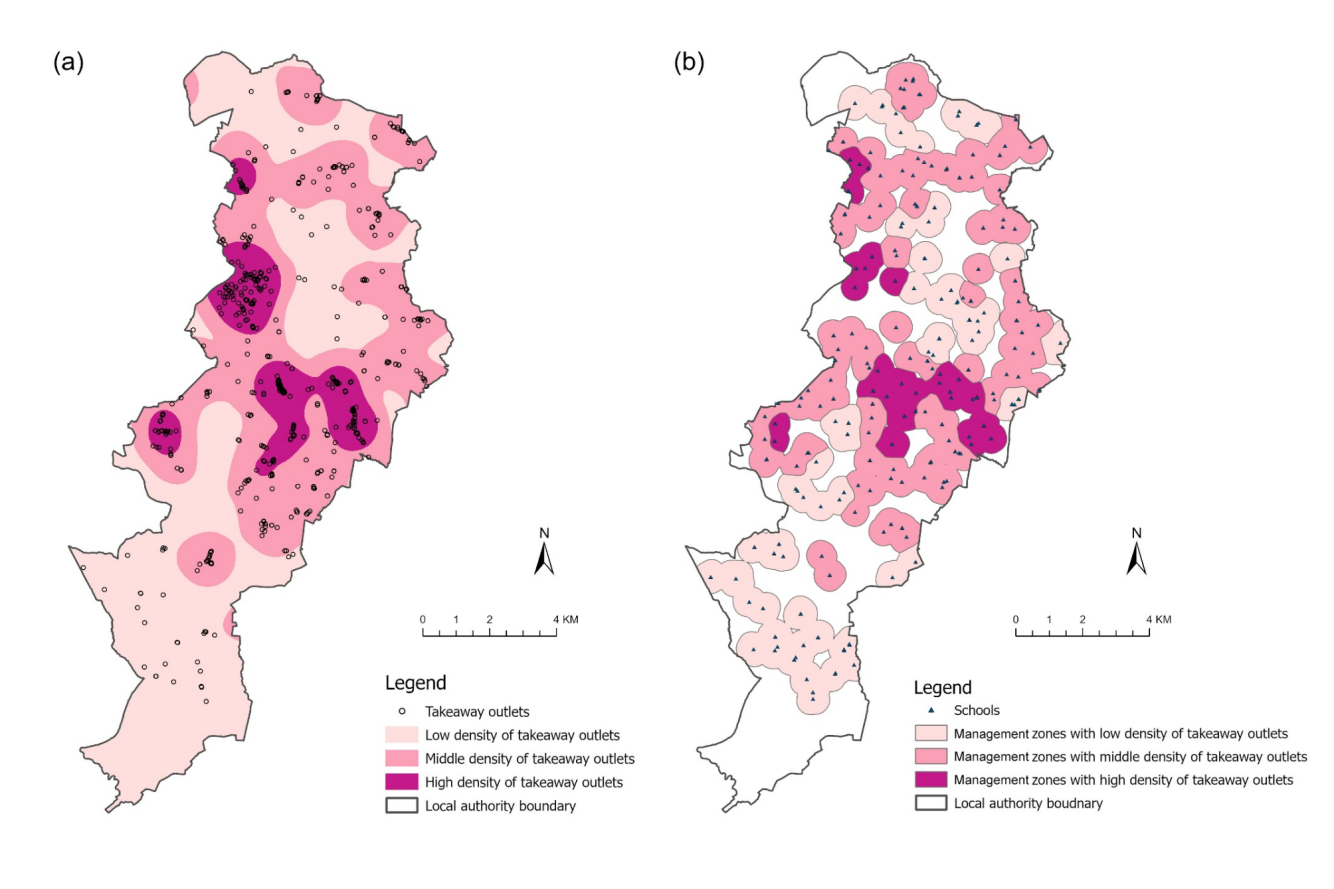
An illustration of the two-step process of deriving non-overlapping management zones with distinct densities of takeaways. Kernel density surface of takeaways in 2015 in Manchester LA **(a)**. Non-overlapping management zones of high, middle, and low takeaway density **(b)**

To address this, we devised an approach that subdivides merged zones into non-overlapping polygons according to takeaway density (Fig. 4). To subdivide zones, we created a kernel density surface of takeaways in 2015. Takeaways within neighbouring LAs, within 2 km of the LA boundary, were considered when creating this density surface in order to minimise boundary distortion. We then classified the kernel density raster cell values into three distinct categories using natural breaks; a technique that pinpoints thresholds to maximize intergroup variance (Fig. 4a). The calculated takeaway density values were assigned to centre points of school sites. Management zones around schools were dissolved where they belonged to the same category of takeaway density. Overlaps within the resulting zones were eliminated using the centre line method. The final non-overlapping management zones in Manchester LA are shown in Fig. 4b. These mutually exclusive, dissolved management zones exhibit heterogeneous counts of takeaways across this LA, which is crucial for avoiding exposure misclassification in the subsequent spatial intersection analysis.

#### Exposure estimation within takeaway management zones

**Fig. 5:**
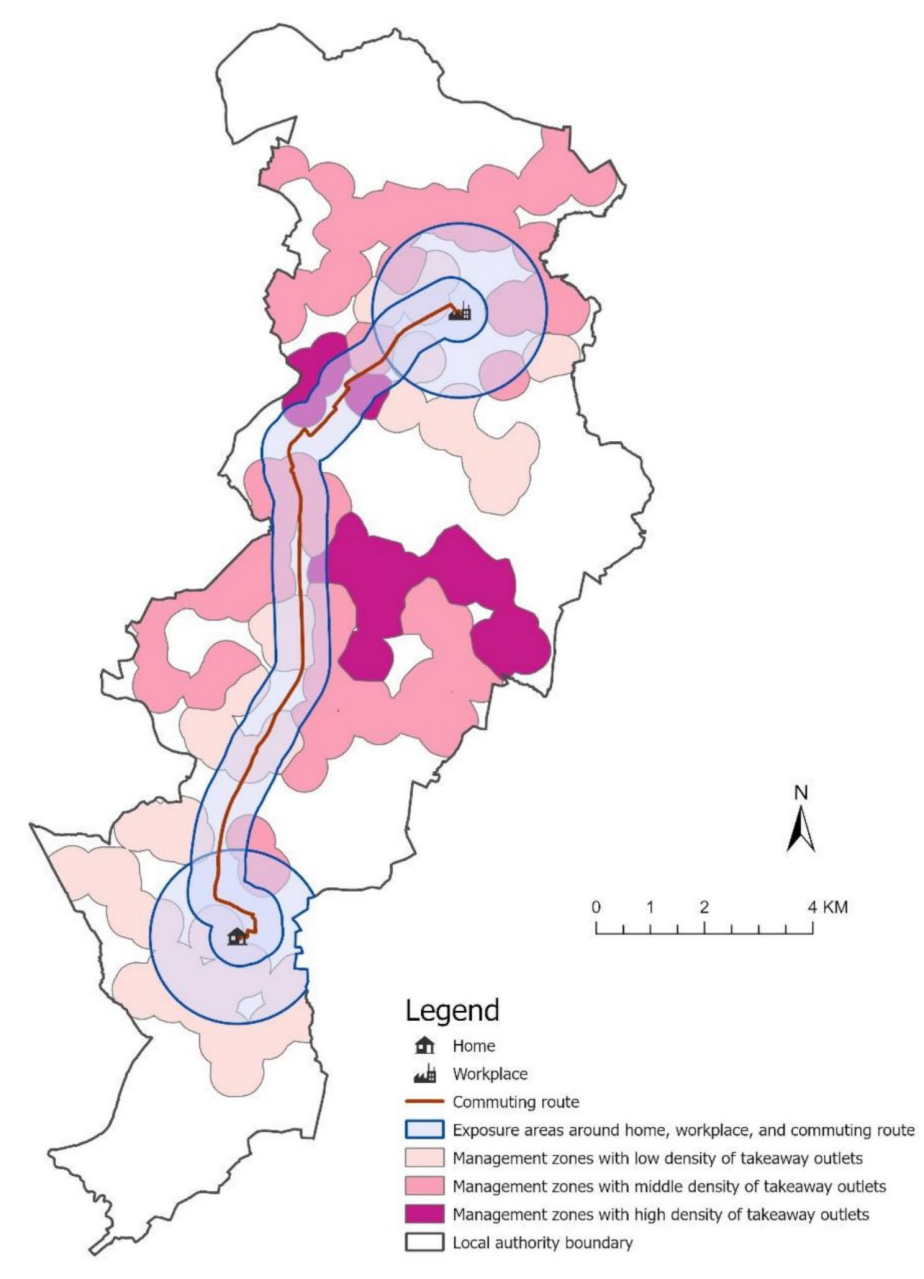
An example intersection of home, work and commuting route domains for a car commuter, with non-overlapping takeaway management zones, in Manchester LA

Having created non-overlapping management zones, we then intersected these with home, work and commuting route domains in order to calculate takeaways exposure. We presumed that the degree of this exposure corresponds to the extent of this overlap, with the assumption of an even distribution of takeaways within non-overlapping management zones (Fig. 5).

Thus, the exposure generated by takeaways in management zone *k* for a sub-population commuting from residential OA *i* to workplace OA *j* using travel mode *m* around their usual place *p* (including domains at home, at work, and along commuting route) is defined as the product of the number of the sub-population, the count of takeaways within management zone *k*, and the ratio of the area where this sub-population’s exposure space intersects management zone *k* to the total area of management zone *k*.

$$\begin{gathered}\:Exposur{e_{ijmkp}} = \hfill \\\,\,\,\,\,\,\,\,\,\,\,\,\,\,\:\frac{{populatio{n_{ijm}} \times \:coun{t_k}\: \times \:area\_intersecte{d_{ijmkp}}}}{{are{a_k}}}\: \hfill \\ \end{gathered}$$

Put simply, if a home, work, commute polygon overlaps half of a management zone, then 50% of the number of takeaways within this zone would be incorporated into the calculation of exposure.

Total population-level exposure to takeaways within management zones in any given LA can therefore be expressed as the sum of $\:{Exposure}_{ijmkp}$:

$$\begin{gathered}\:Exposure\_total = \hfill \\\,\,\,\,\,\,\,\,\,\,\,\sum\nolimits_i {\sum\nolimits_j {\sum\nolimits_m {\sum\nolimits_k {\sum\nolimits_p {Exposur{e_{ijmkp}}} } } } } \: \hfill \\ \end{gathered}$$

Per capita exposure to takeaways refers to the mean count of takeaways within management zones to which an individual in a local authority is exposed across home, work and commuting route domains. It is derived by dividing total exposure by the population count:

$$\:Exposure\_per\:capita=\:\:\frac{Exposure\_total}{population\_total}$$

Spatial analyses for exposure computation were conducted using ArcGIS Pro 3.0 [37].

## Results

For brevity, here we present detailed results for Manchester, an adopter LA of the non-London major urban conurbation RUC and summary findings for other LAs. We present detailed results for the other five LAs in Additional file 3.

### Forecasts of the count of takeaways within management zones to 2031

The ARIMA model variant best suited to the non-adopter, non-London urban with major conurbation RUC to which Manchester belongs, was ARIMA(0,1,0) with drift. Among the candidate models as described in the methods, it demonstrated the lowest AICc value (448.62) after applying first-order differencing, and an absence of autocorrelation (Ljung-Box test p value = 0.93). Furthermore, this ARIMA model variant exhibited superior forecasting accuracy compared to ETS, as evidenced by smaller RMSE (269.84 vs. 346.22 for ETS) and MASE (3.56 vs. 5.04 for ETS) values (Additional files 1 and 2).

**Fig. 6:**
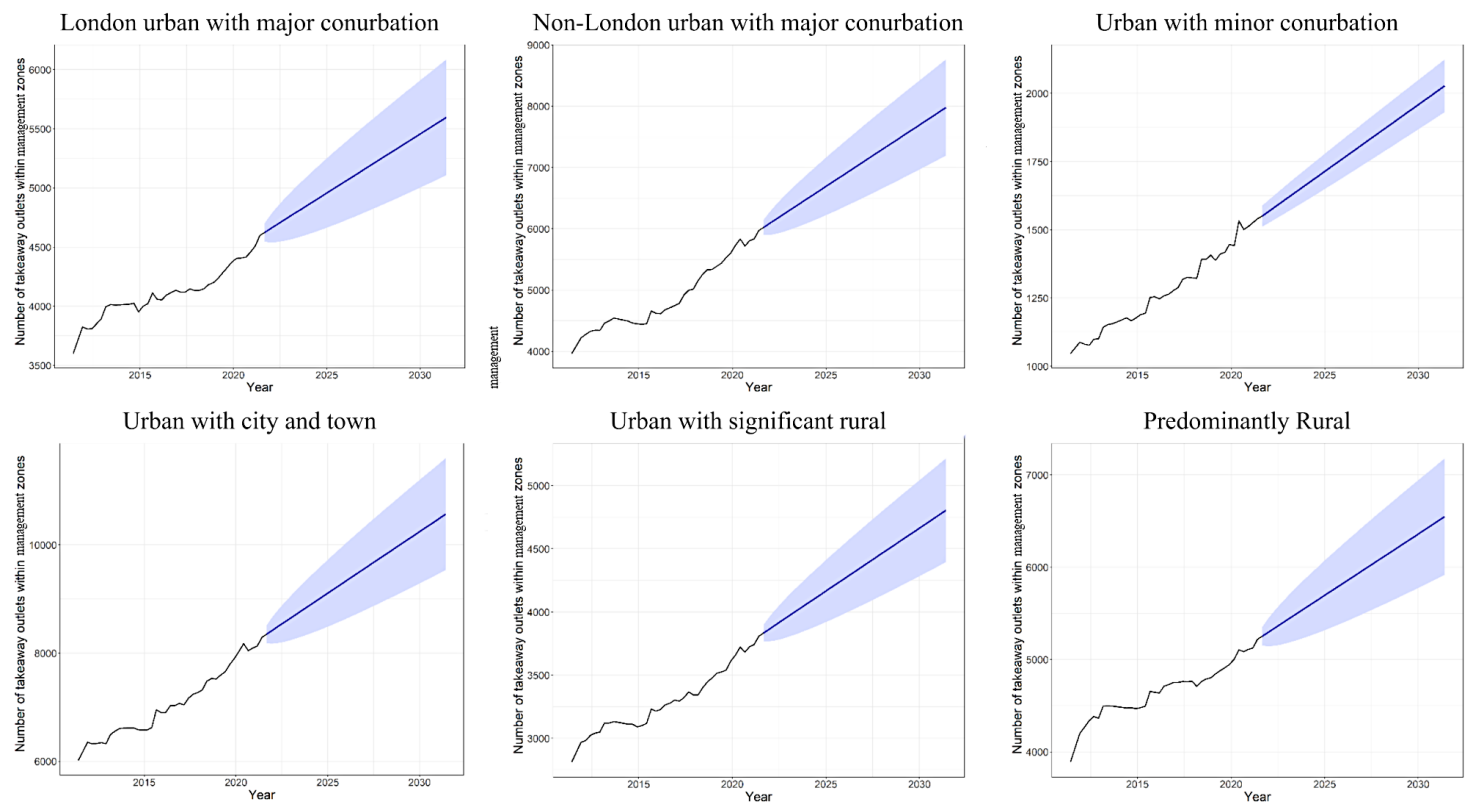
Historic observations (2011–2021) and ten-year ARIMA model forecasts (2021–2031) of the count of takeaways within management zones for non-adopter local authorities across six rural urban classes in England. Blue ribbons are 95% forecast intervals

For these non-adopter LAs, the estimated count of takeaways exhibited an upward trajectory (Fig. 6). The count of takeaways was estimated to be 6173.6 (95% CI: 5925.6, 6421.6) in 2022 and was forecast to increase to 7979.0 (95% CI: 7194.7, 8763.3) by 2031 (Table 2). These figures indicate a cumulative growth from the baseline year of 2015, of 38.7% (95% CI: 33.1%, 44.3%) by 2022 and 79.3% (95% CI: 61.6%, 96.9%) by 2031 (Table 2). This was the strongest forecast growth across all non-adopter LAs by RUC (Fig. 6).

Takeaway counts within management zones in non-adopter LAs across the five other RUC were also forecast to increase from 2022 to 2031, albeit at variable growth rates from 39.4 to 72.3% (Fig. 6). Among these, the urban with minor conurbation class exhibited the next strongest growth to 2031, at 72.3% (95% CI: 64.1%, 80.5%), followed by urban with city and town at 59.7% (95% CI: 44.1%, 75.3%). The London urban with major conurbation class was forecast the weakest growth to 2031 at 39.4% (95% CI: 27.2%, 51.6%). A detailed summary of changes in counts of takeaways and rates of change for these other RUC can be found in Additional file 3.

**Table 2:** Forecasts of counts in numbers of takeaways in management zones around schools, in non-adopter local authorities of the non-London urban with major conurbation rural urban class, to which Manchester belongs, in England. Corresponding rates of change also shown

Year	Count of takeaways			Cumulative rate of change		
	Estimate	Lower bound of 95% forecast interval	Upper bound of 95% forecast interval	Estimate	Lower bound of 95% forecast interval	Upper bound of 95% forecast interval
Baseline year (2015)	4451.0			-		
Forecast						
2022	6173.6	5925.6	6421.6	38.7%	33.1%	44.3%
2023	6374.2	6023.5	6725.0	43.2%	35.3%	51.1%
2024	6574.8	6145.2	7004.4	47.7%	38.1%	57.4%
2025	6775.4	6279.4	7271.4	52.2%	41.1%	63.4%
2026	6976.0	6421.4	7530.6	56.7%	44.3%	69.2%
2027	7176.6	6569.1	7784.1	61.2%	47.6%	74.9%
2028	7377.2	6721.0	8033.4	65.7%	51.0%	80.5%
2029	7577.8	6876.3	8279.3	70.2%	54.5%	86.0%
2030	7778.4	7034.4	8522.5	74.8%	58.0%	91.5%
2031	7979.0	7194.7	8763.3	79.3%	61.6%	96.9%

Note: All values correspond to the second quarter of each calendar year

### Estimates of population-level exposure to takeaways within management zones

RUC-specific growth rates calculated for non-adopter LAs were used to estimate the annual *count* of takeaways within management zones in the six adopter LAs by RUC (see Additional file 4 for estimated counts of takeaways within individual management zones in Manchester). As described in the methods, these forecasts for each LA were used to characterise population-level takeaway exposure, with per capita estimates and forecast intervals shown for Manchester in Table 3. In 2022, mean population exposure per person within management zones and combined home, work and commuting route domains was estimated at 114.6 (95% CI: 110.0, 119.2) takeaways. By 2031, this was forecast to rise to 148.2 (95% CI: 133.6, 162.7) takeaways.

**Table 3:** Forecast population-level per capita exposure to takeaways within management zones and combined home, work and commuting route domains in Manchester LA. Corresponding rates of change also shown

Year	Exposure to takeaways per person			Cumulative rate of change		
	Estimate	Lower bound of 95% forecast interval	Upper bound of 95% forecast interval	Estimate	Lower bound of 95% forecast interval	Upper bound of 95% forecast interval
Baseline year (2015)	82.65			-		
Forecast						
2022	114.63	110.03	119.24	38.7%	33.1%	44.3%
2023	118.36	111.84	124.87	43.2%	35.3%	51.1%
2024	122.08	114.10	130.06	47.7%	38.1%	57.4%
2025	125.81	116.59	135.02	52.2%	41.1%	63.4%
2026	129.53	119.23	139.83	56.7%	44.3%	69.2%
2027	133.25	121.97	144.54	61.2%	47.6%	74.9%
2028	136.98	124.80	149.16	65.7%	51.0%	80.5%
2029	140.70	127.68	153.73	70.2%	54.5%	86.0%
2030	144.43	130.61	158.24	74.8%	58.0%	91.5%
2031	148.15	133.59	162.72	79.3%	61.6%	96.9%

Note: All values correspond to the second quarter of each calendar year

Exposure to takeaways within management zones in our other five adopter LAs also exhibited growth to 2031 (Additional file 5), albeit less strong in absolute terms than in Manchester. Moreover, compared to rural LAs, urban LAs were also forecast stronger growth in absolute exposure. Among urban LAs, Sheffield ranked second to Manchester in estimated change in absolute population exposure, which was forecast to rise from 94.4 to 120.6 (95% CI: 114.8, 126.3) takeaways by 2031. In contrast, the rural LAs of North Somerset and Fenland were forecast to have more modest increases in absolute population exposures, from 22.2 to 27.3 takeaways, and from 20.1 to 24.6 takeaways, respectively.

The forecasts of exposure within management zones can be further analysed by examining separate domain-specific exposures in home and work neighbourhoods, and along commuting routes. For Manchester, growth was forecast in each domain from 2022 to 2031 (Additional file 6), with growth in home and work neighbourhoods, and along commuting routes, contributing 49.7%, 31.4% and 18.9% total forecast growth in exposure, respectively.

## Discussion

In this study, we described and validated a novel two-stage approach for forecasting future takeaway growth and population exposure to takeaways within management zones, in six local authorities in England across the urban/rural spectrum. Our approach to calculating population exposure considered both anticipated future growth in the takeaway retail sector, as well as the home and work locations, and typical commuting patterns, of the populations of these areas. First, we forecast the number of takeaways within management zones of each LA to 2031, using time-series forecasting methods. These forecasts were based on and drew strength from national, RUC-specific, historic data on takeaway growth trends. Second, we translated forecasts in the number of takeaways within management zones into population-level exposures. Across our six LAs, we forecast increasing numbers of takeaways within zones up to 2031, albeit with varying growth rates according to RUC (min–max: 39.4-79.3%). Amongst the six exemplar LAs studied, Manchester, a major urban local authority outside of London, was forecast to have the highest mean population exposure to takeaways within management zones in 2031, with 148 takeaways per person on average across home, work and commuting domains.

### Strengths and applicability of the proposed approach

To our knowledge, this is the first study to forecast nationwide future proliferation of takeaways, including within management zones around schools in England. While the spatial clustering of takeaways near schools has been extensively explored [38, 39], most of these studies have been cross-sectional, with none forecasting future growth. Recent longitudinal studies have started to evaluate the effects of planning policies designed to restrict the opening of new takeaways around schools, demonstrating a decrease in both the number of planning applications [7] and new takeaways [8] in these areas. However, focusing on observed changes over a relatively short period (e.g., up to maximum of six years post-intervention) [7], these studies have not addressed long-term growth of takeaways. This is a critical gap because short-term changes in the absolute number of takeaways in response to management zone interventions are relatively modest. By forecasting long-term changes in takeaway retail within management zones, our study provides an approach to address this knowledge gap, ultimately laying the foundations for analyzing longer-term health impacts of the intervention.

By using a time-series ARIMA forecasting model, we proposed a method to extrapolate future takeaway counts and validated this approach over a ten-year time horizon. Despite increasing uncertainty, this method could be used to forecast growth even further into the future. Furthermore and importantly, this forecasting method has the potential to be adapted and used to forecast takeaway growth in other types of areas, as well as growth in other retail sectors that also demonstrate consistent historic trends that permit time-series modelling.

A crucial component of our proposed approach involved the translation of forecast counts of takeaways within management zones into population exposure to takeaways, which has been positively associated with consumption of takeaway-type food and body weight [3, 40, 41]. To maximise accuracy in these calculations of population-level exposure, our method accounted for important day-to-day geographic contexts experienced by working adults i.e. home, work and commuting domains [42, 43]. This was enabled through the use of travel-to-work data from the UK census. Additionally, we considered ease of access to takeaways by transportation mode through applying buffer radii sensitive to commute mode. This approach enables us to measure exposure to takeaways more accurately within the spaces in which the working population regularly operate.

Our forecasts and associated exposures serve to illustrate how numbers of takeaways are likely to accrue in the long term in the absence of intervention. This information could be used by LAs to enhance decision-making and strengthen the case for adoption and robust implementation of, for example, takeaway management zones around schools. Specifically, claims made by prospective takeaway owners, that the addition of a single new takeaway could not possibly make a measurable impact on population health, often prove compelling. Including at appeal, where those tasked with adjudicating planning disputes have not necessarily trained in public health. However, the impacts of such decisions need to be appreciated over the long term, in terms of how the opening of just a single new takeaway can accrue repeatedly over time, ultimately resulting in a meaningful increase in population exposure. Our results forecast for the first time the expected trajectories of this long-term growth across a range of LAs.

We tested our forecasts in LAs across the urban/rural spectrum in England. This was important because those in policy and practice within LAs have reported using the RUC to identify and learn from other similar LAs [15]. Thereby we increase the generalisability and real-world applicability of our findings. Had we focussed only on urban LAs, the scope for learning among rural LAs might potentially have been deemed limited. Moreover, our use of identical management zones across our six LAs, whilst also matching the most frequently adopted zone specification nationwide (400 m buffers around school site boundaries), also ensured that it was possible to compare forecast population exposures across them. We observed heterogeneity in forecast growth rates and population exposures to takeaways, which to some extent are attributable to varying historic counts of takeaways across RUC. The forecast absolute growth in per person exposure to takeaways to 2031 was strongest for Manchester (114.6 to 148.2 outlets), followed by Sheffield (94.4 to 120.6 outlets). However, all six LAs were forecast to experience changes in population exposure to takeaways that might be considered a risk to public health. In relative terms, these forecast growth rates far exceed official estimates of future population growth from 2022 to 2031, which, amongst our study LAs, are predicted to be strongest for Fenland LA at just 6.3% [44]. There is no current or foreseeable mechanism by which new takeaways can be eliminated once established, which underscores the need for prevention.

### Limitations and future work

Our forecasts of growth in takeaways are subject to several limitations. A critical assumption in our forecasts was that historic growth in numbers of takeaways would persist into the future. However, it is possible that this growth may not be sustainable and that the number of takeaways in some LAs could reach a “saturation point”, where the local market for takeaway businesses constrains growth and their numbers cease to grow. We were unable to predict when such a tipping point might occur, although the growth rates that were forecast were not linear, and instead showed a reducing rate of increase over time consistent with saturation being reached at a future point in time. Nonetheless, by 2031 it seems unlikely that this saturation point would be reached in any of our six LAs. The reported number of takeaways per 100,000 population in the LAs we studied (ranging from 67 per 100,000 in North Somerset to 148 per 100,000 in Blackburn with Darwen) were, even in 2031, considerably lower than the maximum density reported in any LA in England (232 per 100,000 in Blackpool LA) in 2017 [45]. This suggests that there is potential for takeaways to continue proliferating in the future. However, to enhance the accuracy of longer-term forecasting, it would be valuable to identify when, and the market conditions under which this saturation point might be reached, considering changes in population, evolving takeaway business models, and socio-cultural factors that could influence shifts in dietary consumption. A promising direction for future research is to adopt a systems perspective (e.g., using system dynamics modelling), wherein researchers can explore how growth in takeaways is influenced by economic and societal factors over time [46].

Previous research underscores the proliferation of takeaways in new residential and retail areas [47, 48]. However, due to data limitations, our forecasts were not able to explicitly accommodate future developments that might introduce new takeaway retail units. Consequently, our anticipated growth of takeaways in areas poised for commercial and retail development may have been underestimated. We also assumed that commuting patterns would remain stable to 2031. However, the commuting behaviours of future working cohorts may differ from those recorded in the 2011 census, particularly given the shift towards remote working in the post-COVID-19 era [49]. This shift could potentially result in an overestimation of exposure based on the premise of an individual having distinct home and work locations, and a linked commuting exposure. Future studies can incorporate these changes once newer waves of OA-level commuting flow data become available, for example from the 2021 census, which were not available at the time of this study. We were also unable to account for exposure to takeaways through online food delivery services, the use of which constitutes a growing trend among this population. That said, our forecasts are credible, reflecting historic trends of remarkable consistency within RUC, which allowed ARIMA models to have good fit (evidenced by low AICc values). These models also outperformed exponential smoothing in terms of forecast accuracy, as indicated by lower RMSE and MASE scores in time-series cross-validation. Additionally, we did not incorporate *time* spent at home, workplace or on commuting routes into the exposure computation, primarily due to the lack of availability of such data. However, previous epidemiological research in UK populations has also used a simple sum of exposures across these three domains without applying time weights, in relation to diet and health [29].

Our forecasts show heterogeneity in growth rates across six LAs across the urban/rural spectrum. However, our model was not able to forecast within-LA heterogeneity in growth rates. Previous research has suggested that clustering of takeaways around cities and retail cores is evidence of the predominant influence of agglomeration on the locational dynamics of these outlets [47, 50]. Moreover, when the density of existing food outlets exceeds a given threshold, heightened competition can hinder the entry of new outlets [51, 52]. Despite this understanding, there remains a dearth of robust and location-specific evidence regarding the response of takeaway growth to existing outlet density, as well as other geographical factors (e.g. spatial relationships with other types of outlets) and socio-demographic variables (e.g. deprivation), which could enable more accurate forecasting [13]. This presents an area where future studies can contribute valuable insights. One fruitful direction is to develop machine learning-based forecast model that can incorporate diverse predictors and accommodate non-linear predictor-outcome relationships.

Our approach was unable to predict the precise locations of new takeaways, and consequently relied upon the assumption of an even distribution of takeaways within each management zone. This is unlikely to be a realistic assumption, which introduces a degree of uncertainty into our estimations of exposure, stemming from a possible disparity with the actual count of takeaways within the part of a management zone contributing to exposure. Nonetheless, by dividing merged management zones based on the density of existing takeaways, our analysis effectively captures the spatially varying distribution of takeaways at a refined spatial scale. Future studies could explore alternative approaches such as survival models to forecast the future landscape of takeaways, considering specific locations.

## Conclusions

Until now, there were no forecasts of future takeaway growth within takeaway management zones around schools in England, and by extension, no future forecasts of population exposure to takeaways. We developed and validated a novel two-stage approach to address this evidence gap, which currently hinders both research and policymaking. First, we used a time-series model to forecast numbers of takeaways within management zones to 2031, in six local authorities across the urban/rural spectrum. Second, we translated these forecasts of the number of takeaways within management zones into population-level exposures across home, work and commuting domains, which have been positively associated with takeaway-type food consumption and body weight. Across our six LAs, we forecast increasing numbers of takeaways within zones up to 2031, albeit with varying growth rates by RUC (min–max: 39.4-79.3%), and strongest growth in Manchester (classified as a non-London urban with major conurbation LA). All six LAs were forecast changes in population exposure within management zones to takeaways that might be considered a risk to public health, which suggests that planning restrictions to limit proliferation may be a helpful public health intervention over the long term. Our novel approach offers a promising avenue for understanding future trends in takeaway retail outlets within management zones and population-level exposure to them, with flexibility to model other types of retail outlet trends and for other areas.

## Electronic Supplementary Material

Below is the link to the electronic supplementary material.

Supplementary Material 1

Supplementary Material 2

Supplementary Material 3

Supplementary Material 4

Supplementary Material 5

Supplementary Material 6

## Data Availability

The data that support the findings of this study are available from the Office for National Statistics (UK census travel to work data) and Ordnance Survey (Points of interest, school locations data), but restrictions apply to the availability of these data. These data may be available from the data providers upon request.
