# Supplementary material for: Development of an approach to forecast future takeaway outlet growth around schools and population exposure to takeaways in England
Source: Int J Health Geogr. 2024 Nov 10;23:24. doi: 10.1186/s12942-024-00383-6 (PMC11550555; doi:10.1186/s12942-024-00383-6)
Supplement: Supplementary file 1 — Supplementary Material 1 [file 12942_2024_383_MOESM1_ESM.docx]

# Additional file 1: Explanations, forecasts and accuracy metrics of ETS model

The ETS model, an extensively employed statistical methodology for time series forecast, encapsulates three critical components, namely: Error, Trend, and Seasonality (R. J. Hyndman et al., 2002). The error component accounts for the random variability within the time series data, which remains unexplained by the trend and seasonal components. It can be represented either additively (A) or multiplicatively (M). The trend component captures the consistent change in the level of the time series over the course of time. This can be modelled as absent (N), additive (A), or multiplicative (M). In an additive damped (Ad) trend model, the trend gradually levels off as time progresses. The damping factor governs the rate at which the trend approaches a horizontal asymptote. Lastly, the seasonality component is capable of capturing seasonal fluctuations. This can be modelled as absent (N), additive (A), or multiplicative (M). The model parameters are estimated using the maximum likelihood estimation (MLE) method. The best model is chosen by minimizing a bias-corrected version of Akaike's Information Criterion (AICc) (R. Hyndman & Athanasopoulos, 2018). The process of ETS modelling was executed using the “forecast” package (R. Hyndman et al., 2023; R. J. Hyndman & Khandakar, 2008) in R.

ETS (Exponential Smoothing) was chosen as the alternative method for sensitivity analysis because of its simplicity and robustness in handling various types of time-series data. ETS is particularly effective when the data shows a clear trend, which aligns well with the observed growth in the number of takeaway outlets over time. Additionally, ETS is a well-established method in time-series analysis, providing a reliable benchmark for comparison with the more complex ARIMA models. By choosing ETS, we ensured that our sensitivity analysis utilized a widely recognized and accessible method, making the results both practical and interpretable.

Figure 1.1 and Tables 1.1 present the forecasted yearly counts of takeaways within hypothetical exclusion zones (EZs) in non-implementer local authorities (LAs) across six rural-urban classifications in England. Table 2.2 presents the accuracy metrics derived from the time series cross-validation.


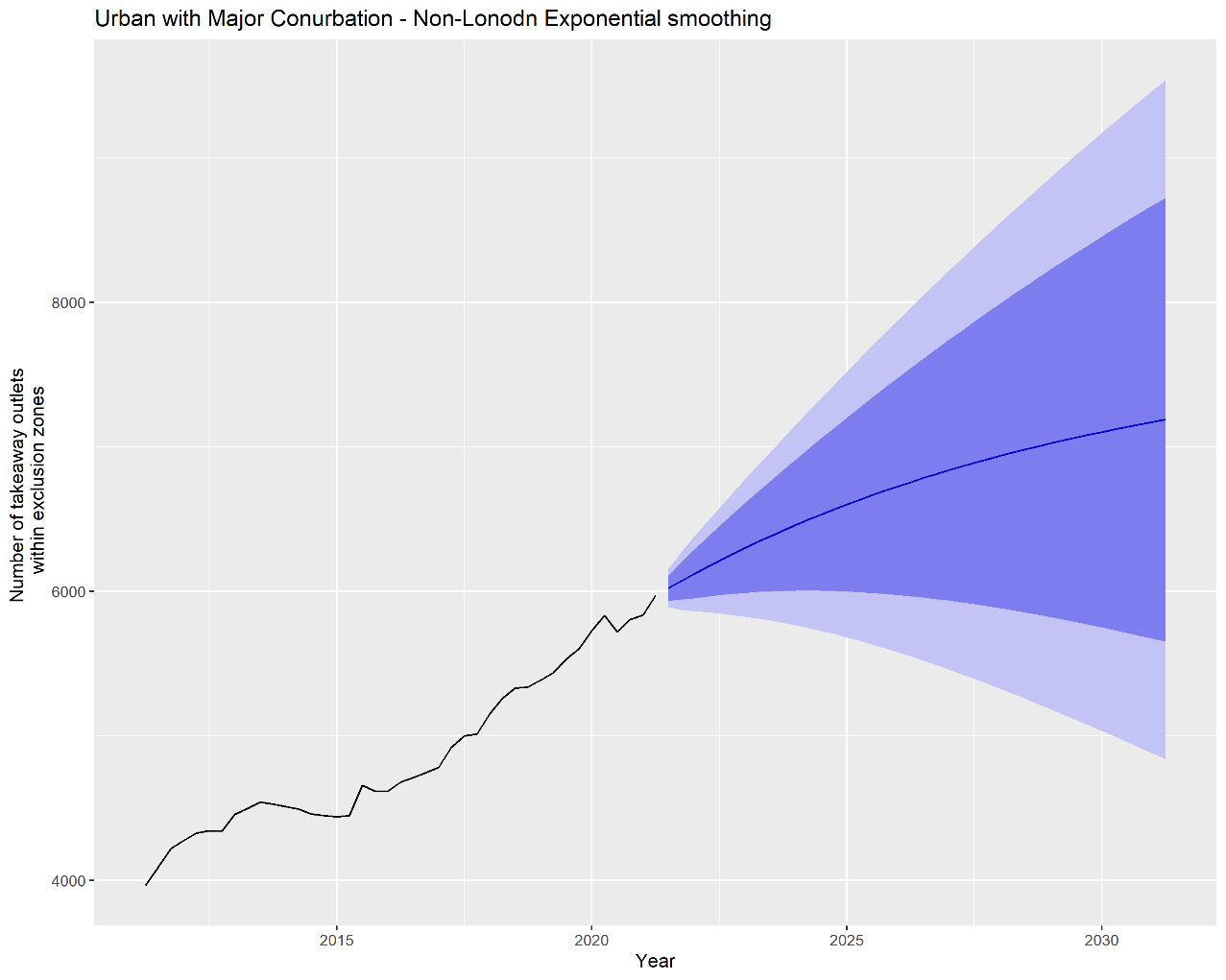


Figure 1.1. Historical observations (from June 2011 to June 2021) and ten-year ETS model forecasts (from September 2021 to June 2031) of the count of takeaway outlets within exclusion zones for non-implementer Non-London Urban with Major Conurbation local authorities. The 95% and 80 % prediction intervals are illustrated by the dark and light lavender blue ribbons, respectively.

Table 1.1. Forecasts of absolute count of takeaway outlets in non-implementer LAs of the Urban with Major Conurbation Non-London category using the ETS model.

|  | Point.Forecast | Lo.80 | Hi.80 | Lo.95 | Hi.95 |
| --- | --- | --- | --- | --- | --- |
| 2021 Q3 | 6022.09 | 5935.24 | 6108.94 | 5889.27 | 6154.91 |
| 2021 Q4 | 6071.96 | 5942.98 | 6200.95 | 5874.70 | 6269.23 |
| 2022 Q1 | 6120.37 | 5953.64 | 6287.10 | 5865.38 | 6375.37 |
| 2022 Q2 | 6167.36 | 5964.45 | 6370.26 | 5857.04 | 6477.68 |
| 2022 Q3 | 6212.96 | 5974.47 | 6451.45 | 5848.22 | 6577.70 |
| 2022 Q4 | 6257.22 | 5983.31 | 6531.14 | 5838.30 | 6676.14 |
| 2023 Q1 | 6300.19 | 5990.79 | 6609.59 | 5827.01 | 6773.37 |
| 2023 Q2 | 6341.89 | 5996.85 | 6686.93 | 5814.20 | 6869.58 |
| 2023 Q3 | 6382.36 | 6001.48 | 6763.25 | 5799.85 | 6964.88 |
| 2023 Q4 | 6421.65 | 6004.68 | 6838.62 | 5783.95 | 7059.35 |
| 2024 Q1 | 6459.78 | 6006.50 | 6913.07 | 5766.54 | 7153.02 |
| 2024 Q2 | 6496.79 | 6006.97 | 6986.62 | 5747.67 | 7245.91 |
| 2024 Q3 | 6532.72 | 6006.14 | 7059.29 | 5727.39 | 7338.04 |
| 2024 Q4 | 6567.58 | 6004.06 | 7131.10 | 5705.76 | 7429.41 |
| 2025 Q1 | 6601.43 | 6000.79 | 7202.06 | 5682.83 | 7520.02 |
| 2025 Q2 | 6634.27 | 5996.37 | 7272.18 | 5658.68 | 7609.87 |
| 2025 Q3 | 6666.16 | 5990.85 | 7341.47 | 5633.36 | 7698.95 |
| 2025 Q4 | 6697.10 | 5984.28 | 7409.93 | 5606.93 | 7787.27 |
| 2026 Q1 | 6727.14 | 5976.71 | 7477.57 | 5579.45 | 7874.83 |
| 2026 Q2 | 6756.29 | 5968.18 | 7544.41 | 5550.98 | 7961.61 |
| 2026 Q3 | 6784.59 | 5958.74 | 7610.44 | 5521.56 | 8047.62 |
| 2026 Q4 | 6812.06 | 5948.43 | 7675.68 | 5491.26 | 8132.86 |
| 2027 Q1 | 6838.72 | 5937.29 | 7740.14 | 5460.11 | 8217.32 |
| 2027 Q2 | 6864.59 | 5925.36 | 7803.82 | 5428.17 | 8301.02 |
| 2027 Q3 | 6889.71 | 5912.68 | 7866.73 | 5395.48 | 8383.94 |
| 2027 Q4 | 6914.08 | 5899.28 | 7928.88 | 5362.08 | 8466.09 |
| 2028 Q1 | 6937.74 | 5885.20 | 7990.29 | 5328.02 | 8547.47 |
| 2028 Q2 | 6960.71 | 5870.47 | 8050.95 | 5293.33 | 8628.09 |
| 2028 Q3 | 6983.00 | 5855.12 | 8110.88 | 5258.05 | 8707.95 |
| 2028 Q4 | 7004.63 | 5839.18 | 8170.09 | 5222.22 | 8787.05 |
| 2029 Q1 | 7025.63 | 5822.68 | 8228.59 | 5185.87 | 8865.39 |
| 2029 Q2 | 7046.02 | 5805.65 | 8286.38 | 5149.04 | 8942.99 |
| 2029 Q3 | 7065.80 | 5788.11 | 8343.48 | 5111.75 | 9019.85 |
| 2029 Q4 | 7085.00 | 5770.10 | 8399.90 | 5074.03 | 9095.97 |
| 2030 Q1 | 7103.64 | 5751.63 | 8455.65 | 5035.92 | 9171.36 |
| 2030 Q2 | 7121.73 | 5732.73 | 8510.73 | 4997.43 | 9246.02 |
| 2030 Q3 | 7139.29 | 5713.42 | 8565.16 | 4958.60 | 9319.97 |
| 2030 Q4 | 7156.33 | 5693.71 | 8618.95 | 4919.45 | 9393.21 |
| 2031 Q1 | 7172.87 | 5673.64 | 8672.10 | 4880.00 | 9465.74 |
| 2031 Q2 | 7188.93 | 5653.23 | 8724.63 | 4840.28 | 9537.58 |

Table 1.2. Accuracy metrics for ETS(A,Ad,N) model at various forecasting horizons for Non-London Urban with Major Conurbation non-implementer local authorities.

| h | ME | RMSE | MAE | MPE | MAPE | MASE | RMSSE | ACF1 |
| --- | --- | --- | --- | --- | --- | --- | --- | --- |
| 1 | 16.27 | 72.02 | 53.93 | 0.30 | 1.06 | 0.85 | 0.90 | 0.05 |
| 2 | 31.43 | 108.03 | 93.44 | 0.57 | 1.85 | 1.48 | 1.35 | 0.54 |
| 3 | 49.26 | 146.91 | 132.09 | 0.89 | 2.62 | 2.09 | 1.83 | 0.64 |
| 4 | 67.64 | 181.75 | 159.53 | 1.23 | 3.19 | 2.53 | 2.27 | 0.76 |
| 5 | 101.67 | 219.18 | 190.10 | 1.84 | 3.80 | 3.01 | 2.74 | 0.81 |
| 6 | 138.15 | 261.96 | 233.66 | 2.49 | 4.63 | 3.70 | 3.27 | 0.77 |
| 7 | 176.86 | 298.38 | 273.18 | 3.19 | 5.34 | 4.33 | 3.72 | 0.85 |
| 8 | 219.50 | 346.22 | 318.42 | 3.96 | 6.15 | 5.04 | 4.32 | 0.80 |
