# Supplementary material for: Development of an approach to forecast future takeaway outlet growth around schools and population exposure to takeaways in England
Source: Int J Health Geogr. 2024 Nov 10;23:24. doi: 10.1186/s12942-024-00383-6 (PMC11550555; doi:10.1186/s12942-024-00383-6)
Supplement: Supplementary file 2 — Supplementary Material 2 [file 12942_2024_383_MOESM2_ESM.docx]

**Additional file 2: Parameters, residual plots and accuracy metrics of ARIMA model**

Table 2.1. Parameters and bias-corrected Akaike information criterion (AICc) values for ARIMA models chosen for each rural-urban class.

| Rural-urban classification | Model chosen | AICc |
| --- | --- | --- |
| London urban with major conurbation | ARIMA(0,1,0) with drift | 410.86 |
| Non-London urban with major conurbation | ARIMA(0,1,0) with drift | 448.62 |
| Urban with minor conurbation | ARIMA(0,1,1) with drift | 357.17 |
| Urban with city and town | ARIMA(0,1,0) with drift | 470.59 |
| Urban with significant rural | ARIMA(0,1,0) with drift | 396.72 |
| Largely or mainly rural | ARIMA(0,1,0) with drift | 1103.05 |

**Plots of residuals**


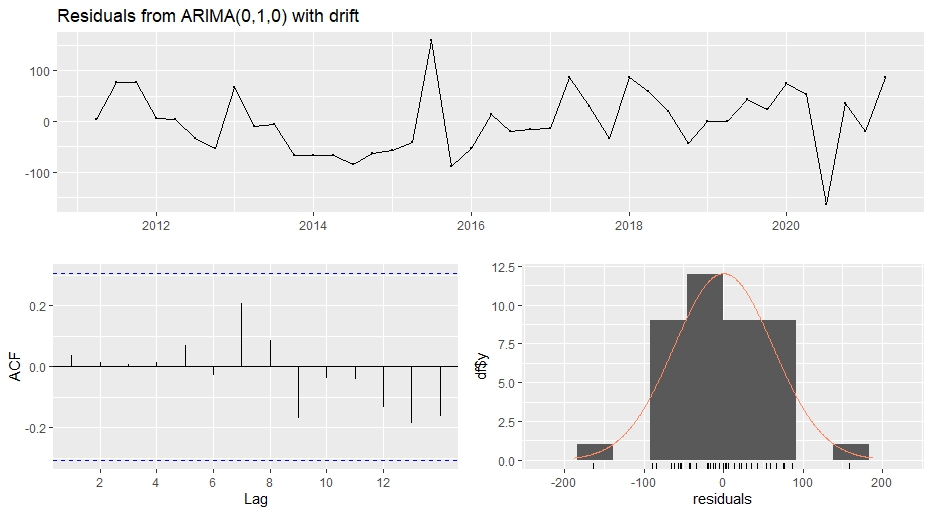


Figure 2.1. The plot of residuals (upper), the ACF plot of residuals (bottom left), and the distribution plot of residuals of the ARIMA(0,1,0) with drift model for Non-London Urban with Major Conurbation non-implementer local authorities.

**Accuracy metrics**

Table 2.2. Accuracy metrics for ARIMA(0,1,0) with drift model at various forecasting horizons for Non-London Urban with Major Conurbation non-implementer local authorities.

| h | ME | RMSE | MAE | MPE | MAPE | MASE | RMSSE | ACF1 |
| --- | --- | --- | --- | --- | --- | --- | --- | --- |
| 1 | 1.13 | 66.45 | 51.86 | -0.02 | 1.04 | 0.82 | 0.83 | 0.04 |
| 2 | -0.20 | 98.62 | 83.94 | -0.09 | 1.70 | 1.33 | 1.23 | 0.54 |
| 3 | -0.90 | 129.37 | 111.99 | -0.15 | 2.27 | 1.77 | 1.61 | 0.70 |
| 4 | -0.85 | 159.22 | 127.72 | -0.19 | 2.62 | 2.02 | 1.99 | 0.80 |
| 5 | 7.68 | 189.77 | 146.94 | -0.08 | 3.01 | 2.33 | 2.37 | 0.83 |
| 6 | 17.07 | 219.93 | 177.63 | 0.04 | 3.61 | 2.81 | 2.75 | 0.81 |
| 7 | 26.95 | 243.60 | 198.82 | 0.20 | 4.01 | 3.15 | 3.04 | 0.84 |
| 8 | 38.71 | 269.84 | 224.99 | 0.39 | 4.49 | 3.56 | 3.37 | 0.82 |
