# Supplementary material for: Development of an approach to forecast future takeaway outlet growth around schools and population exposure to takeaways in England
Source: Int J Health Geogr. 2024 Nov 10;23:24. doi: 10.1186/s12942-024-00383-6 (PMC11550555; doi:10.1186/s12942-024-00383-6)
Supplement: Supplementary file 3 — Supplementary Material 3 [file 12942_2024_383_MOESM3_ESM.docx]

**Additional file 3: Forecasts of counts and corresponding change rates of takeaway outlets within exclusion zones in non-adopter local authorities across rural-urban classes**

Table 3.1. Forecasts of absolute count and corresponding change rate of takeaway outlets in non-adopter LAs of the London Urban with Major Conurbation category.

| Year | Count of takeaways | | | Cumulative rate of change | | | Year-over-year annual growth rate |
| --- | --- | --- | --- | --- | --- | --- | --- |
|  |  |  |  |  |  |  |  |
|  | Estimate | Lower bound of 95% prediction interval | Upper bound of 95% prediction interval | Estimate | Lower bound of 95% prediction interval | Upper bound of 95% prediction interval |  |
| Base year | 4014.00 | |  | - |  |  |  |
| (2013) |  |  |  |  |  |  |  |
| Forecast |  |  |  |  |  |  |  |
| 2022 | 4698.80 | 4544.08 | 4853.52 | 17.1% | 13.2% | 20.9% | 2.17% |
| 2023 | 4798.60 | 4579.80 | 5017.40 | 19.5% | 14.1% | 25.0% | 2.12% |
| 2024 | 4898.40 | 4630.42 | 5166.38 | 22.0% | 15.4% | 28.7% | 2.08% |
| 2025 | 4998.20 | 4688.76 | 5307.64 | 24.5% | 16.8% | 32.2% | 2.04% |
| 2026 | 5098.00 | 4752.04 | 5443.96 | 27.0% | 18.4% | 35.6% | 2.00% |
| 2027 | 5197.80 | 4818.82 | 5576.78 | 29.5% | 20.1% | 38.9% | 1.96% |
| 2028 | 5297.60 | 4888.26 | 5706.94 | 32.0% | 21.8% | 42.2% | 1.92% |
| 2029 | 5397.40 | 4959.79 | 5835.01 | 34.5% | 23.6% | 45.4% | 1.88% |
| 2030 | 5497.20 | 5033.05 | 5961.35 | 37.0% | 25.4% | 48.5% | 1.85% |
| 2031 | 5597.00 | 5107.74 | 6086.26 | 39.4% | 27.2% | 51.6% | 1.82% |

Table 3.2. Forecasts of absolute count and corresponding change rate of takeaway outlets in non-adopter LAs of the Urban with Minor Conurbation category.

| Year | Count of takeaways | | | Cumulative rate of change | | | Year-over-year annual growth rate |
| --- | --- | --- | --- | --- | --- | --- | --- |
|  | Estimate | Lower bound of 95% prediction interval | Upper bound of 95% prediction interval | Estimate | Lower bound of 95% prediction interval | Upper bound of 95% prediction interval |  |
| Base year | 1177.0 |  |  | - |  |  |  |
| (2014) |  |  |  |  |  |  |  |
| Forecast |  |  |  |  |  |  |  |
| 2022 | 1587.50 | 1541.72 | 1633.28 | 34.9% | 31.0% | 38.8% | 3.02% |
| 2023 | 1636.43 | 1582.60 | 1690.27 | 39.0% | 34.5% | 43.6% | 3.08% |
| 2024 | 1685.37 | 1624.53 | 1746.20 | 43.2% | 38.0% | 48.4% | 2.99% |
| 2025 | 1734.30 | 1667.20 | 1801.41 | 47.3% | 41.6% | 53.1% | 2.90% |
| 2026 | 1783.24 | 1710.40 | 1856.08 | 51.5% | 45.3% | 57.7% | 2.82% |
| 2027 | 1832.17 | 1754.02 | 1910.33 | 55.7% | 49.0% | 62.3% | 2.74% |
| 2028 | 1881.10 | 1797.97 | 1964.23 | 59.8% | 52.8% | 66.9% | 2.67% |
| 2029 | 1930.04 | 1842.21 | 2017.86 | 64.0% | 56.5% | 71.4% | 2.60% |
| 2030 | 1978.97 | 1886.69 | 2071.25 | 68.1% | 60.3% | 76.0% | 2.54% |
| 2031 | 2027.91 | 1931.38 | 2124.44 | 72.3% | 64.1% | 80.5% | 2.47% |

Table 3.3. Forecasts of absolute count and corresponding change rate of takeaway outlets in non-adopter LAs of the Urban with City and Town category.

| Year | Count of takeaways | | | Cumulative rate of change | | | Year-over-year annual growth rate |
| --- | --- | --- | --- | --- | --- | --- | --- |
|  |  |  |  |  |  |  |  |
|  | Estimate | Lower bound of 95% prediction interval | Upper bound of 95% prediction interval | Estimate | Lower bound of 95% prediction interval | Upper bound of 95% prediction interval |  |
| Base year | 6618.00 |  |  | - |  |  |  |
| (2014) |  |  |  |  |  |  |  |
| Forecast |  |  |  |  |  |  |  |
| 2022 | 8520.30 | 8193.86 | 8846.74 | 28.7% | 23.8% | 33.7% | 2.74% |
| 2023 | 8747.60 | 8285.95 | 9209.25 | 32.2% | 25.2% | 39.2% | 2.67% |
| 2024 | 8974.90 | 8409.49 | 9540.31 | 35.6% | 27.1% | 44.2% | 2.60% |
| 2025 | 9202.20 | 8549.33 | 9855.07 | 39.0% | 29.2% | 48.9% | 2.53% |
| 2026 | 9429.50 | 8699.57 | 10159.43 | 42.5% | 31.5% | 53.5% | 2.47% |
| 2027 | 9656.80 | 8857.20 | 10456.40 | 45.9% | 33.8% | 58.0% | 2.41% |
| 2028 | 9884.10 | 9020.43 | 10747.77 | 49.4% | 36.3% | 62.4% | 2.35% |
| 2029 | 10111.40 | 9188.10 | 11034.70 | 52.8% | 38.8% | 66.7% | 2.30% |
| 2030 | 10338.70 | 9359.39 | 11318.01 | 56.2% | 41.4% | 71.0% | 2.25% |
| 2031 | 10566.00 | 9533.72 | 11598.28 | 59.7% | 44.1% | 75.3% | 2.20% |

Table 3.4. Forecasts of absolute count and corresponding change rate of takeaway outlets in non-adopter LAs of the Urban with Significant Rural category.

| Year | Count of takeaways | | | Cumulative rate of change | | | Year-over-year annual growth rate |
| --- | --- | --- | --- | --- | --- | --- | --- |
|  |  |  |  |  |  |  |  |
|  | Estimate | Lower bound of 95% prediction interval | Upper bound of 95% prediction interval | Estimate | Lower bound of 95% prediction interval | Upper bound of 95% prediction interval |  |
| Base year | 3112.00 |  |  | - |  |  |  |
| (2014) |  |  |  |  |  |  |  |
| Forecast |  |  |  |  |  |  |  |
| 2022 | 3908.60 | 3778.95 | 4038.25 | 25.6% | 21.4% | 29.8% | 2.61% |
| 2023 | 4008.20 | 3824.85 | 4191.55 | 28.8% | 22.9% | 34.7% | 2.55% |
| 2024 | 4107.80 | 3883.24 | 4332.36 | 32.0% | 24.8% | 39.2% | 2.48% |
| 2025 | 4207.40 | 3948.10 | 4466.70 | 35.2% | 26.9% | 43.5% | 2.42% |
| 2026 | 4307.00 | 4017.09 | 4596.91 | 38.4% | 29.1% | 47.7% | 2.37% |
| 2027 | 4406.60 | 4089.02 | 4724.18 | 41.6% | 31.4% | 51.8% | 2.31% |
| 2028 | 4506.20 | 4163.18 | 4849.22 | 44.8% | 33.8% | 55.8% | 2.26% |
| 2029 | 4605.80 | 4239.09 | 4972.51 | 48.0% | 36.2% | 59.8% | 2.21% |
| 2030 | 4705.40 | 4316.45 | 5094.35 | 51.2% | 38.7% | 63.7% | 2.16% |
| 2031 | 4805.00 | 4395.01 | 5214.99 | 54.4% | 41.2% | 67.6% | 2.12% |

Table 3.5. Forecasts of absolute count and corresponding change rate of takeaway outlets in non-adopter LAs of the Largely or Mainly Rural category.

| Year | Count of takeaways | | | Cumulative rate of change | | | Year-over-year annual growth rate |
| --- | --- | --- | --- | --- | --- | --- | --- |
|  |  |  |  |  |  |  |  |
|  | Estimate | Lower bound of 95% prediction interval | Upper bound of 95% prediction interval | Estimate | Lower bound of 95% prediction interval | Upper bound of 95% prediction interval |  |
| Base year | 4475.0 | |  | - |  |  |  |
| (2014) |  |  |  |  |  |  |  |
| Forecast |  |  |  |  |  |  |  |
| 2022 | 5351.80 | 5152.65 | 5550.95 | 19.6% | 15.1% | 24.0% | 2.54% |
| 2023 | 5484.60 | 5202.95 | 5766.25 | 22.6% | 16.3% | 28.9% | 2.48% |
| 2024 | 5617.40 | 5272.46 | 5962.34 | 25.5% | 17.8% | 33.2% | 2.42% |
| 2025 | 5750.20 | 5351.89 | 6148.51 | 28.5% | 19.6% | 37.4% | 2.36% |
| 2026 | 5883.00 | 5437.68 | 6328.32 | 31.5% | 21.5% | 41.4% | 2.31% |
| 2027 | 6015.80 | 5527.97 | 6503.63 | 34.4% | 23.5% | 45.3% | 2.26% |
| 2028 | 6148.60 | 5621.69 | 6675.51 | 37.4% | 25.6% | 49.2% | 2.21% |
| 2029 | 6281.40 | 5718.11 | 6844.69 | 40.4% | 27.8% | 53.0% | 2.16% |
| 2030 | 6414.20 | 5816.74 | 7011.66 | 43.3% | 30.0% | 56.7% | 2.11% |
| 2031 | 6547.00 | 5917.22 | 7176.78 | 46.3% | 32.2% | 60.4% | 2.07% |
