# Supplementary material for: Development of an approach to forecast future takeaway outlet growth around schools and population exposure to takeaways in England
Source: Int J Health Geogr. 2024 Nov 10;23:24. doi: 10.1186/s12942-024-00383-6 (PMC11550555; doi:10.1186/s12942-024-00383-6)
Supplement: Supplementary file 4 — Supplementary Material 4 [file 12942_2024_383_MOESM4_ESM.docx]

**Additional file 4: Forecasts of counts of takeaway outlets within exclusion zones in Manchester**

Table 4.1. Forecasts of counts of takeaway outlets within exclusion zones in Manchester.

| Index | Count of takeaway outlets in the base year of 2015 | Estimated count of takeaway outlets in 2031 | Lower bound of 95% prediction interval | Upper bound of 95% prediction interval |
| --- | --- | --- | --- | --- |
| manchester_1 | 2 | 3.59 | 3.23 | 3.94 |
| manchester_2 | 23 | 41.23 | 37.18 | 45.28 |
| manchester_3 | 0 | 0.00 | 0.00 | 0.00 |
| manchester_4 | 2 | 3.59 | 3.23 | 3.94 |
| manchester_5 | 0 | 0.00 | 0.00 | 0.00 |
| manchester_6 | 2 | 3.59 | 3.23 | 3.94 |
| manchester_7 | 0 | 0.00 | 0.00 | 0.00 |
| manchester_8 | 1 | 1.79 | 1.62 | 1.97 |
| manchester_9 | 7 | 12.55 | 11.31 | 13.78 |
| manchester_10 | 1 | 1.79 | 1.62 | 1.97 |
| manchester_11 | 3 | 5.38 | 4.85 | 5.91 |
| manchester_12 | 1 | 1.79 | 1.62 | 1.97 |
| manchester_13 | 16 | 28.68 | 25.86 | 31.50 |
| manchester_14 | 13 | 23.30 | 21.01 | 25.59 |
| manchester_15 | 57 | 102.18 | 92.14 | 112.22 |
| manchester_16 | 2 | 3.59 | 3.23 | 3.94 |
| manchester_17 | 3 | 5.38 | 4.85 | 5.91 |
| manchester_18 | 20 | 35.85 | 32.33 | 39.38 |
| manchester_19 | 4 | 7.17 | 6.47 | 7.88 |
| manchester_20 | 1 | 1.79 | 1.62 | 1.97 |
| manchester_21 | 33 | 59.16 | 53.34 | 64.97 |
| manchester_22 | 8 | 14.34 | 12.93 | 15.75 |
| manchester_23 | 3 | 5.38 | 4.85 | 5.91 |
| manchester_24 | 70 | 125.48 | 113.15 | 137.82 |
| manchester_25 | 16 | 28.68 | 25.86 | 31.50 |
| manchester_26 | 25 | 44.82 | 40.41 | 49.22 |
| manchester_27 | 127 | 227.66 | 205.29 | 250.04 |
| manchester_28 | 7 | 12.55 | 11.31 | 13.78 |
| manchester_29 | 51 | 91.42 | 82.44 | 100.41 |
| manchester_30 | 17 | 30.47 | 27.48 | 33.47 |
