# Supplementary material for: Development of an approach to forecast future takeaway outlet growth around schools and population exposure to takeaways in England
Source: Int J Health Geogr. 2024 Nov 10;23:24. doi: 10.1186/s12942-024-00383-6 (PMC11550555; doi:10.1186/s12942-024-00383-6)
Supplement: Supplementary file 5 — Supplementary Material 5 [file 12942_2024_383_MOESM5_ESM.docx]

**Additional file 5: Estimated exposure to takeaway outlets within exclusion zones in the six selected adopter local authorities**

Table 5.1. Estimated exposure per working adult aged 16 and above to takeaway outlets within exclusion zones in six local authorities.

| Year | Wandsworth (n=131,356) | | | Manchester (n=189,337) | | | Sheffield (n=220,233) | | |
| --- | --- | --- | --- | --- | --- | --- | --- | --- | --- |
|  | Estimate | Lower bound of 95% prediction interval | Upper bound of 95% prediction interval | Estimate | Lower bound of 95% prediction interval | Upper bound of 95% prediction interval | Estimate | Lower bound of 95% prediction interval | Upper bound of 95% prediction interval |
| 2022 | 85.8 | 83.0 | 88.6 | 114.6 | 110.0 | 119.2 | 94.4 | 91.7 | 97.1 |
| 2023 | 87.6 | 83.6 | 91.6 | 118.4 | 111.8 | 124.9 | 97.3 | 94.1 | 100.5 |
| 2024 | 89.5 | 84.6 | 94.4 | 122.1 | 114.1 | 130.1 | 100.2 | 96.6 | 103.8 |
| 2025 | 91.3 | 85.6 | 96.9 | 125.8 | 116.6 | 135.0 | 103.1 | 99.1 | 107.1 |
| 2026 | 93.1 | 86.8 | 99.4 | 129.5 | 119.2 | 139.8 | 106.0 | 101.7 | 110.3 |
| 2027 | 94.9 | 88.0 | 101.8 | 133.3 | 122.0 | 144.5 | 108.9 | 104.3 | 113.6 |
| 2028 | 96.7 | 89.3 | 104.2 | 137.0 | 124.8 | 149.2 | 111.8 | 106.9 | 116.8 |
| 2029 | 98.6 | 90.6 | 106.6 | 140.7 | 127.7 | 153.7 | 114.7 | 109.5 | 120.0 |
| 2030 | 100.4 | 91.9 | 108.9 | 144.4 | 130.6 | 158.2 | 117.7 | 112.2 | 123.1 |
| 2031 | 102.2 | 93.3 | 111.1 | 148.2 | 133.6 | 162.7 | 120.6 | 114.8 | 126.3 |
| Growth rate from 2022 to 2031 | 19.1% |  |  | 29.2% |  |  | 27.7% |  |  |
| *Year* | *Blackburn with Darwen (n=54,353)* | | | *North Somerset (n=86,990)* | | | *Fenland (n=39,732)* | | |
|  | Estimate | Lower bound of 95% prediction interval | Upper bound of 95% prediction interval | Estimate | Lower bound of 95% prediction interval | Upper bound of 95% prediction interval | Estimate | Lower bound of 95% prediction interval | Upper bound of 95% prediction interval |
| 2022 | 80.8 | 77.7 | 83.9 | 22.2 | 21.5 | 23.0 | 20.1 | 19.4 | 20.9 |
| 2023 | 82.9 | 78.6 | 87.3 | 22.8 | 21.8 | 23.8 | 20.6 | 19.6 | 21.7 |
| 2024 | 85.1 | 79.7 | 90.5 | 23.4 | 22.1 | 24.6 | 21.1 | 19.8 | 22.4 |
| 2025 | 87.3 | 81.1 | 93.5 | 23.9 | 22.5 | 25.4 | 21.6 | 20.1 | 23.1 |
| 2026 | 89.4 | 82.5 | 96.3 | 24.5 | 22.9 | 26.1 | 22.1 | 20.5 | 23.8 |
| 2027 | 91.6 | 84.0 | 99.2 | 25.1 | 23.3 | 26.9 | 22.6 | 20.8 | 24.5 |
| 2028 | 93.7 | 85.5 | 101.9 | 25.6 | 23.7 | 27.6 | 23.1 | 21.2 | 25.1 |
| 2029 | 95.9 | 87.1 | 104.6 | 26.2 | 24.1 | 28.3 | 23.6 | 21.5 | 25.8 |
| 2030 | 98.0 | 88.8 | 107.3 | 26.8 | 24.6 | 29.0 | 24.1 | 21.9 | 26.4 |
| 2031 | 100.2 | 90.4 | 110.0 | 27.3 | 25.0 | 29.7 | 24.6 | 22.3 | 27.0 |
| Growth rate from 2022 to 2031 | 24.0% |  |  | 22.9% |  |  | 22.3% |  |  |
