# Supplementary material for: Development of an approach to forecast future takeaway outlet growth around schools and population exposure to takeaways in England
Source: Int J Health Geogr. 2024 Nov 10;23:24. doi: 10.1186/s12942-024-00383-6 (PMC11550555; doi:10.1186/s12942-024-00383-6)
Supplement: Supplementary file 6 — Supplementary Material 6 [file 12942_2024_383_MOESM6_ESM.docx]

**Additional file 6: Estimated exposure to takeaway outlets within exclusion zones in the domains of the home, workplace, and commuting route in Manchester**

Table 6.1. Estimated exposure per working adult aged 16 and above (n=189,337) to takeaway outlets within hypothetical exclusion zones in Manchester.

| Year | Estimated total exposure per person to takeaway outlets | | | Estimated exposure per person to takeaway outlets around home locations | | | Estimated exposure per person to takeaway outlets around workplace locations | | | Estimated exposure per person to takeaway outlets around commuting routes | | |
| --- | --- | --- | --- | --- | --- | --- | --- | --- | --- | --- | --- | --- |
|  | Estimate | Lower bound of 95% prediction interval | Upper bound of 95% prediction interval | Estimate | Lower bound of 95% prediction interval | Upper bound of 95% prediction interval | Estimate | Lower bound of 95% prediction interval | Upper bound of 95% prediction interval | Estimate | Lower bound of 95% prediction interval | Upper bound of 95% prediction interval |
| 2022 | 114.6 | 110.0 | 119.2 | 57.0 | 54.7 | 59.3 | 36.0 | 34.5 | 37.4 | 21.6 | 20.8 | 22.5 |
| 2023 | 118.4 | 111.8 | 124.9 | 58.9 | 55.6 | 62.1 | 37.1 | 35.1 | 39.2 | 22.3 | 21.1 | 23.6 |
| 2024 | 122.1 | 114.1 | 130.1 | 60.7 | 56.8 | 64.7 | 38.3 | 35.8 | 40.8 | 23.1 | 21.5 | 24.6 |
| 2025 | 125.8 | 116.6 | 135.0 | 62.6 | 58.0 | 67.2 | 39.5 | 36.6 | 42.4 | 23.8 | 22.0 | 25.5 |
| 2026 | 129.5 | 119.2 | 139.8 | 64.4 | 59.3 | 69.6 | 40.6 | 37.4 | 43.9 | 24.5 | 22.5 | 26.4 |
| 2027 | 133.3 | 122.0 | 144.5 | 66.3 | 60.7 | 71.9 | 41.8 | 38.3 | 45.3 | 25.2 | 23.0 | 27.3 |
| 2028 | 137.0 | 124.8 | 149.2 | 68.1 | 62.1 | 74.2 | 43.0 | 39.2 | 46.8 | 25.9 | 23.6 | 28.2 |
| 2029 | 140.7 | 127.7 | 153.7 | 70.0 | 63.5 | 76.5 | 44.1 | 40.1 | 48.2 | 26.6 | 24.1 | 29.0 |
| 2030 | 144.4 | 130.6 | 158.2 | 71.8 | 65.0 | 78.7 | 45.3 | 41.0 | 49.6 | 27.3 | 24.7 | 29.9 |
| 2031 | 148.2 | 133.6 | 162.7 | 73.7 | 66.5 | 80.9 | 46.5 | 41.9 | 51.1 | 28.0 | 25.2 | 30.7 |
